# Identification of a Novel Non-desmoglein Autoantigen in Pemphigus Vulgaris

**DOI:** 10.3389/fimmu.2019.01391

**Published:** 2019-06-19

**Authors:** Giulia Di Lullo, Valentina Calabresi, Feliciana Mariotti, Giovanna Zambruno, Antonio Lanzavecchia, Giovanni Di Zenzo

**Affiliations:** ^1^Tumor Immunology Unit, Division of Immunology, Transplantation and Infectious Diseases, Istituto di Ricovero e Cura a Carattere Scientifico (IRCCS) San Raffaele Scientific Institute, Milan, Italy; ^2^Laboratory of Molecular and Cell Biology, IDI-IRCCS, Rome, Italy; ^3^Genetic and Rare Diseases Research Division, Bambino Gesù Children's Hospital, IRCCS, Rome, Italy; ^4^Institute for Research in Biomedicine, Università della Svizzera italiana, Bellinzona, Switzerland

**Keywords:** pemphigus vulgaris, non-desmoglein autoantigens, autoantibodies, memory B cells, α-catenin, skin, mucous membranes

## Abstract

Pemphigus vulgaris (PV) is an autoimmune bullous disease of the skin and mucous membranes characterized by the presence of circulating and tissue-bound autoantibodies against keratinocyte cell surface antigens, specifically desmoglein (Dsg) 1 and 3. The pathogenic role of anti-Dsg antibodies is well-established, while the mechanism of blister formation is only partly defined. We have applied a previously developed method for the efficient immortalization of IgG+ memory B cells to identify novel target antigens in PV. A human monoclonal antibody reactive with a hitherto unreported non-Dsg antigen was isolated. Immunoprecipitation and immunoblotting studies with keratinocyte extracts indicated α-catenin as the putative antigen, then confirmed by immunoblotting on the recombinant protein. Four of ten PV sera reacted with recombinant α-catenin. Although the isolated human monoclonal antibody was *per se* unable to dissociate keratinocyte monolayers and also to synergize with a pathogenic antibody *in vitro*, further studies are warranted to assess its possible *in vivo* contribution in the multifactorial pathogenesis and heterogeneous manifestations of PV disease.

## Introduction

Pemphigus vulgaris (PV) is a rare but highly disabling and, if untreated, almost always fatal immunobullous disease of the skin and mucous membranes. PV is characterized histologically by loss of cell-cell adhesion between suprabasal keratinocytes, leading to acantholysis, and immunopathologically by the presence of circulating and tissue-bound autoantibodies (autoAbs) against keratinocyte cell surface antigens, specifically desmoglein (Dsg) 1 and 3. PV is considered as a paradigmatic organ-specific autoimmune disease in view of (i) present knowledge of disease autoantigens and pathogenesis and (ii) reproducibility of major clinical and pathogenic features in animal models (1). The existence of both pathogenic and non-pathogenic anti-Dsg autoAbs has recently been underscored by isolation of human monoclonal antibodies (hMabs) from pemphigus patients. Anti-Dsg hMabs characterization has shown that their pathogenic potential primarily depends on the targeted epitopes (1). We have been interested in characterizing the repertoire of naturally occurring autoreactive epithelium-specific memory B cells in pemphigus vulgaris patients. In a first work, we focused on autoantibodies targeting Dsg3 (2). However, (i) the lack of tight correlation between anti-Dsg autoAb titers and disease activity in some patients and (ii) the significant degree of disease heterogeneity point at the importance of non-Dsg autoAbs, that have been progressively, even though not exhaustively, described (3, 4). In fact, besides Dsg3 and Dsg1, other non-desmoglein autoAbs, either pathogenic or non-pathogenic, have been identified in pemphigus patients. AutoAbs endowed with an acantholytic potential target desmocollin 3, α-acetylcholine receptor, pemphaxin, and keratinocyte mitochondria (5–8). On the other hand, the pathogenic role of autoAbs recognizing other autoantigens, such as ATP2C1, desmocollin 1, BP230, periplakin, E-cadherin, desmoglein 4, desmoplakin 1, and desmoplakin 2, remains to be demonstrated (9). In line with this interest, our current work aimed to identify autoAbs targeting non-Dsg membrane-bound or membrane-associated intracellular antigens.

In the present study, we report on the characterization of a hMab isolated from a PV patient and directed to a novel non-Dsg antigen. The hMab reacts with α-catenin that is recognized by almost half of PV sera analyzed.

## Materials and Methods

### Patients, Sera and Isolation of hMabs From a PV Patient

Peripheral blood was obtained from 2 patients (PVC and PVF) suffering from active mucocutaneous PV. The patients showed typical clinical, histological, and immunopathological features and had high-titer anti-Dsg circulating autoantibodies (PVC: Dsg3, 127 U/ml, Dsg1, 90 U/ml; PVF: Dsg3, 191 U/ml, Dsg1, 170 U/ml), as assessed by ELISA kits based on ectodomain of Dsg1 and Dsg3 (MBL, Nagoya, Japan). hMabs were isolated by a highly-efficient protocol for the immortalization of IgG+ memory B cell with Epstein Barr virus (EBV) in the presence of a Toll-like receptor agonist, as previously described (2). In detail, IgG+ memory B cells were isolated from cryopreserved peripheral blood mononuclear cells using anti-CD22 microbeads (Miltenyi Biotec, Bo, Italy) followed by depletion of cells carrying IgM, IgD, and IgA by cell sorting. Multiple replicate microcultures of 10–30 IgG+ memory B cells/well (for a total of 2 to 8 ×10^4^ purified cells) were infected with EBV and CpG as previously described (10). Culture supernatants were tested for binding to Dsg1- and Dsg3-coated ELISA plates and for binding to HaCaT keratinocyte cell line monolayers (both on live cells and on fixed and permeabilized cells) by immunofluorescence (IF) assay using an automated fluorescence microscope (Pathway 855, BD). The specificity of positive polyclonal cultures was further assessed by IF on primary human keratinocytes. Positive reactivities were confirmed by the propagation of oligoclonal cultures. Positive cultures were cloned by limiting dilution and expanded; antibodies were purified using protein G columns. Serum samples were collected from 10 PV and 16 bullous pemphigoid (BP) patients and 10 healthy donors. This study was carried out in conformity with the Helsinki guidelines and with approval of the IDI-IRCCS Ethics Committee. All the biological samples were obtained after patient's informed consent.

### Immunofluorescence Analyses

IF studies were performed according to the procedure described in Di Zenzo et al. with minor modifications (2). Briefly, supernatants from immortalized human memory B cells were screened on monolayers of live and fixed/permeabilized HaCaT cells. After washing with phosphate-buffered saline, cells were stained with Alexa Fluor 488–conjugated goat anti-human IgG (Invitrogen, Carlsbad, CA, USA). The isolated monoclonal antibodies were further tested on permeabilized HaCaT cells and on primary keratinocytes. Non-keratinocyte cell lines, i.e., MRC9, Hela, and SKMEL cells, were used as controls for IF analyses on cell monolayers. Human antibodies of irrelevant specificity were used as negative controls. Serial images of stained keratinocyte monolayers were acquired by the BD Pathway 855 automated fluorescence microscope. Staining was also performed on cryo sections of normal human skin, guinea pig and monkey esophagus and revealed with fluorescein isothiocyanate-conjugated anti-human IgG antibody (Agilent DAKO, Santa Clara, CA, USA).

### Immunoprecipitation and Immunoblotting Analyses

Immunoprecipitation (IP) of ^35^S-labeled keratinocyte extracts by hMab PVF144 was carried out as previously described (11). The precipitated proteins, separated on sodium dodecyl sulfate-polyacrylamide gel electrophoresis on 6% gels under reducing conditions, were detected by autoradiography. Immunoblotting (IB) experiments on keratinocyte extracts were performed as previously reported using both horseradish peroxidase (HRP)-conjugated and alkaline phosphatase-conjugated secondary antibodies (12). Bands were quantified using ImageJ Software (National Institutes of Health, Bethesda, MD, USA) and their intensities were normalized respect to the positive control signal obtained by anti-α-catenin antibody. The cut-off value was set as the medium value +2 standard deviations obtained measuring signals obtained with healthy donors. Commercial primary antibodies were purchased from Progen Biotechnik GmbH (Heidelberg, Germany) (anti-desmocollin 2), BD Biosciences (San Jose, CA, USA; anti-α-catenin), and Santa Cruz Biotechnology, Inc (Dallas, TX, USA; anti-γ-catenin, anti-β-catenin, anti-p120, and anti-E-cadherin). Recombinant tagged human α1-catenin was purchased from Abcam (Cambridge, UK). To rule out that PV sera were mainly reacting against the fused glutathione S-transferase (GST) moiety of the α-catenin recombinant protein, IB experiments by using commercial tagged protein and equimolar GST were performed (data not shown).

### Enzyme Linked ImmunoSorbent Assay (ELISA)

Briefly, recombinant Dsg1 and Dsg3 ectodomains were produced in baculovirus and used for coating of ELISA plates. The plates were, then, blocked with 1% bovine serum albumin and incubated with antibodies followed by HRP-conjugated anti-human IgG (Jackson ImmunoResearch, Baltimore, PA, USA) (2).

### Keratinocyte Dissociation Assay

The assay was performed as previously reported (13). Briefly, primary human keratinocyte cells were seeded onto 12-well plates and 24 h post-confluence treated with monoclonal antibodies. After adding exfoliative toxin A to cleave Dsg1 protein, the cell monolayers were detached with dispase I (Merck, KGaA, Darmstadt, Germany) and subjected to mechanical stress by pipetting. The monolayer fragments, fixed by adding a 3% formaldehyde solution, were subsequently stained using crystal violet. To investigate a possible synergistic effect PVF144 was applied to the monolayer together with a pathogenic antibody (PVB28) (2) at optimal (4 μg/ml) and suboptimal concentrations (1 μg/ml and 0.25 μg/ml; **Figure 4**) and a non-pathogenic antibody (2, PVB28 in a germline version that is not able to dissociate the keratinocyte monolayer; data not shown).

## Results

### Isolation of hMabs Specific for Non-Dsg Epithelial Antigens in Pemphigus Patients

**Figure 1:**
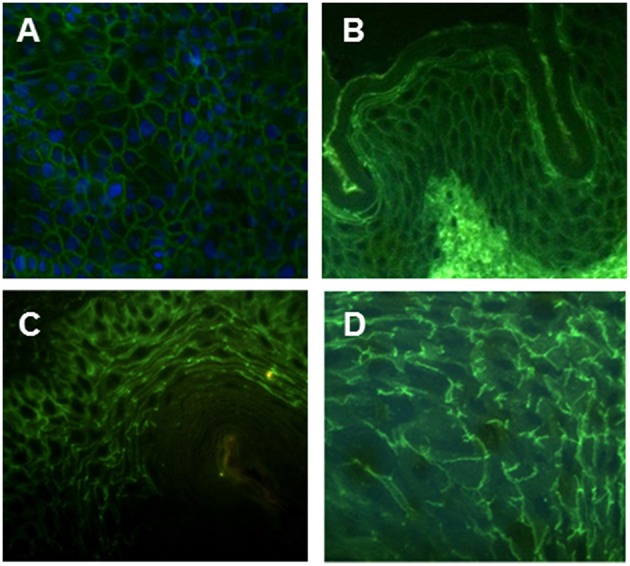
Intercellular staining pattern of hMab PVF144. PVF144 binds a membrane associated epithelial antigen showing a typical intercellular staining pattern on permeabilized HaCaT keratinocytes (nuclear counterstain is obtained with DAPI) (20X) **(A)**, human skin (40X) **(B)** guinea pig (40X) **(C)**, and monkey esophagus (40X) **(D)**.

In order to identify hMabs targeting non-Dsg membrane-bound or membrane-associated intracellular antigens, we took advantage of the same strategy by which we had previously isolated and finely characterized several Dsg-reactive PV patient-derived monoclonal autoantibodies (2). In detail, peripheral blood samples were collected from 2 patients with mucocutaneous PV: one with long-lasting steroid-resistant disease (PVC) and the other prior to treatment initiation (PVF). IgG+ memory B cells were isolated by magnetic and fluorescence-activated cell sorting, seeded in 96-well microplates, and immortalized with EBV in the presence of irradiated mononuclear cells and oligodeoxynucleotides containing CpG motifs, as previously described (10). The reactivity of antibodies secreted in the supernatants of growing polyclonal cultures was screened by IF staining both on live and on fixed and permeabilized cells from the human keratinocyte HaCaT cell line. The polyclonal antibodies produced by the vast majority of isolated cultures bound to surface antigens on the keratinocyte membrane and were reactive with Dsg1 and/or Dsg3 ectodomains by ELISA, as previously reported (2, and data not shown). Besides such prevalent pattern of reactivity, supernatants of rare cultures from both patients showed a distinctive membrane-associated fishnet reactivity, detected only on permeabilized keratinocytes, and were negative in ELISA on Dsg1 and Dsg3 ectodomains. The human Mabs PVF144 (IgG1-isotype), PVC6 (IgG1-isotype), and PVC33 (isotype IgG3-isotype) were cloned by limiting dilution and showed the same IF pattern as the original polyclonal cultures also on permeabilized HaCaT keratinocytes (Figure 1A), suggesting their specificity for a membrane-associated intracellular (i.e., non-exposed) autoantigen. In addition, they showed an intercellular staining pattern by IF on human skin, guinea pig, and monkey esophagus (Figures 1B–D), very similar to IF staining pattern of anti-Dsg3 antibodies (2, and data not shown). Supernatants from the selected clones were tested on non-keratinocyte cell lines, i.e., MRC-9 (fibroblast), SKMEL (melanoma), and Hela (epithelial) cells: they failed to stain both live and permeabilized cells, apart from a reactivity on permeabilized Hela cells (data not shown), hinting at a specificity for membrane-associated intracellular antigens expressed on keratinocytes and other epithelial cells.

### Identification of the Intracellular Epithelial Target Antigen of hMab PVF144

**Figure 2:**
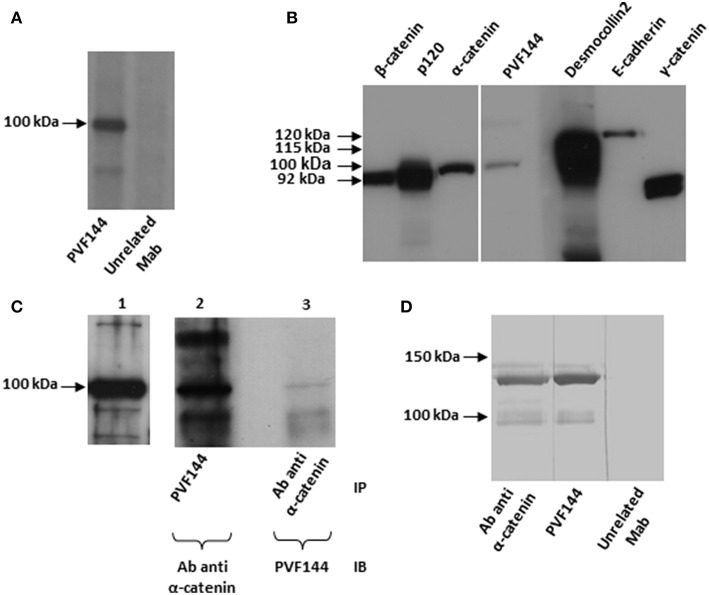
PVF144 binds a membrane-associated epithelial antigen: α-catenin. PVF144 immunoprecipitates (IP) an unknown antigen of 100 kDa from radiolabeled normal human keratinocyte extracts **(A)**. Immunoblotting (IB) experiments on keratinocyte extracts by using PVF144 and commercial antibodies suggest that α-catenin could be the putative antigen of 100 kDa: PVF144 and a monoclonal murine anti-α-catenin antibody (Ab) react with a keratinocyte antigen of similar molecular weight (100 kDa) **(B)**. Anti-α-catenin commercial antibody reacts to α-catenin from keratinocyte extracts by IB (lane 1); PVF144 immunoprecipitates α-catenin recognized by the commercial anti-α-catenin antibody by IB (lane 2) and, viceversa, anti-α-catenin antibody immunoprecipitates α-catenin recognized by PVF144 by IB (lane 3). The faint reactivity observed in lane 3 could be related to the epitope recognized **(C)**. IB performed with a recombinant GST-tagged human α-catenin (120 kDa) confirms that the target of PVF144 is α-catenin. The lower bands are likely degradation products **(D)**.

IP studies with the 3 selected clones on radiolabelled keratinocyte extracts to identify the target antigen showed reactivity to a 100 kDa antigen for PVF144 (Figure 2A) and to 190–210 kDa antigens for PVC6 and PVC33, respectively (data not shown). As the IP results from the latter two clones pointed at members of the plakin-family as candidate target antigens and autoreactivity of PV sera to plakin family members has been already documented (14, 15), we chose to further characterize PVF144 with the aim to identify a possible novel membrane-associated PV autoantigen. Subsequent IB experiments using commercial antibodies reacting with keratinocyte membrane-associated proteins with a molecular weight of ~100 kDa (desmocollin 2–115 kDa; α-catenin-102 kDa; E-cadherin-120 kDa; β-catenin-92 kDa; γ-catenin-83 kDa; P120-catenin 80–100 kDa) indicated α-catenin as the putative antigen (Figure 2B). Sequential IP and IB experiments further supported α-catenin as the target antigen of PVF144. Specifically, a protein immunoprecipitated from keratinocyte extracts with PVF144 was recognized by a commercial anti-α-catenin antibody, and an anti-α-catenin antibody was in turn able to immunoprecipitate a protein recognized by PVF144 in IB (Figure 2C). Finally, IB experiments with the recombinant tagged protein unequivocally confirmed the specific binding of PVF144 to α-catenin (Figure 2D).

### α-Catenin Is Recognized by PV Patient Sera

**Figure 3:**
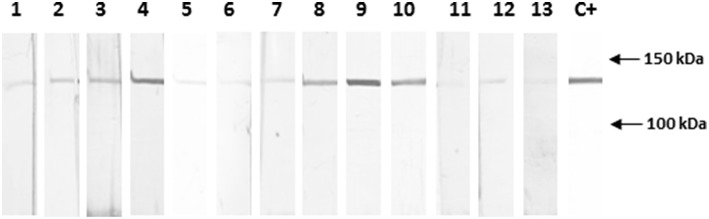
Almost half of PV sera specifically react with recombinant α-catenin. Immunoblotting with sera obtained from 10 pemphigus vulgaris (PV) patients shows that 4 of 10 sera (4, 8, 9, 10) react with the novel epithelial antigen, while 3 healthy donor sera (11, 12, 13), representative of 10 sera analyzed, show only a background signal. The positive control (C+) is the commercial anti-α-catenin antibody. The background signal, could be also due to reactivity to the tag protein (GST). Quantification and normalization of bands using ImageJ analysis (data not shown) have confirmed the reported results (see Materials and Methods section).

To determine whether autoAbs of the same specificity as PVF144 were present in the sera of PV patients, we performed IB experiments with 10 PV sera on recombinant tagged α-catenin (Figure 3). Four out of 10 PV patients (40%) showed the same reactivity of PVF144 to α-catenin, whereas the other PV sera and 10 normal human control sera showed only a faint signal, indicating that this autoreactivity was well-represented in PV patients, even though not shared by all patients. In order to evaluate whether this reactivity is disease-specific, 16 BP patient sera were analyzed on recombinant α-catenin by IB. Only one of 16 BP (7 representative BP sera shown and other 9 not shown) reacted to α-catenin (Supplementary Figure 1) underlining the specificity of this autoantigen. The weak signals found in the other remaining PV, PB, and healthy donor sera might be ascribed to reactivity to the tag protein (GST).

### PVF144 Is Not Able to Dissociate a Keratinocyte Monolayer and to Synergize With a Human Pathogenic Antibody

**Figure 4:**
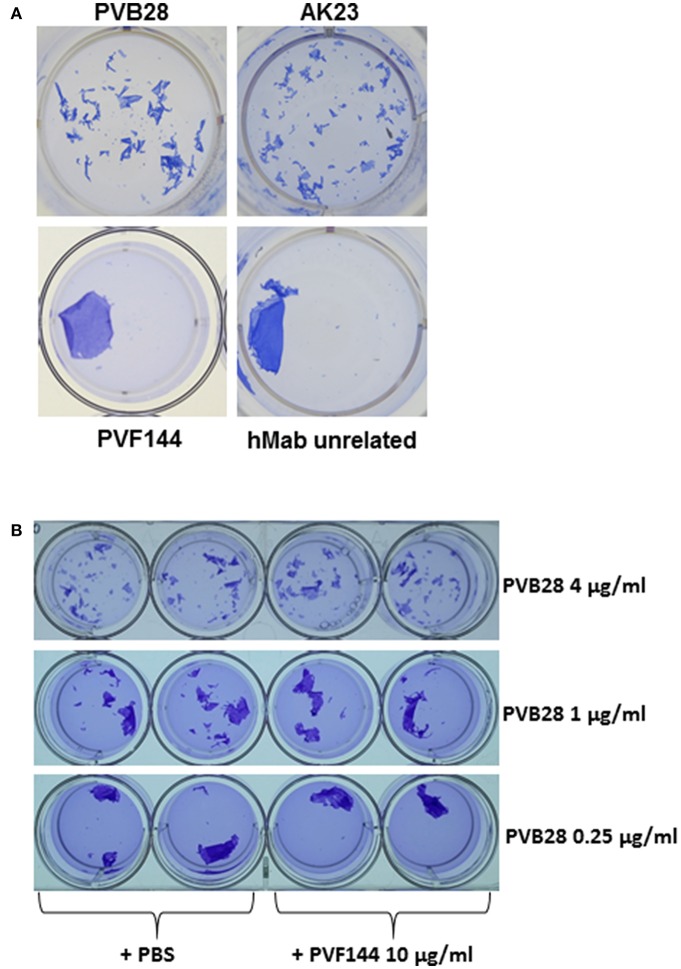
PVF144 is not able to dissociate a keratinocyte monolayer even in the presence of suboptimal concentrations of PVB28. A representative keratinocyte dissociation experiment. Primary human keratinocytes, seeded to confluence, were incubated with PVF144, an unrelated human Mab (negative control) and, as positive controls, the human pathogenic Mab PVB28 (7) and the murine pathogenic Mab AK23 (14), both Dsg3-specific **(A)**. To investigate synergistic potential of cloned antibody we have employed PVF144 (10 μg/ml) together with optimal (4 μg/ml) and suboptimal concentrations (1 μg/ml and 0.25 μg/ml) of the pathogenic anti-Dsg3 antibody PVB28 without obtaining any difference in ability of PVB28, with or without PVF144, to dissociate the keratinocyte monolayer **(B)**.

The presence of anti-α-catenin autoAbs in several PV sera raised the question as to their pathogenic potential. Previous studies showed the ability of intact autoAbs to enter the cytosol or nucleus of living cells (16, 17). More recently, Marchenko et al. reported that PV autoAbs could penetrate keratinocytes and react with intracellular mitochondrial proteins (8). These findings suggested that a hMab to an intracellular antigen could exert its pathogenic ability on live keratinocytes. Thus, an *in vitro* dissociation assay was used to evaluate the pathogenic activity of the hMab PVF144. This approach allows to measure the ability of a specific antibody or a mixture of antibodies, such as a serum, to fragment a monolayer of primary human keratinocytes seeded to confluence. The keratinocyte monolayers, incubated for 24 h with PVF144, remained intact, similarly to monolayers incubated with an unrelated hMab used as negative control (Figure 4). As expected, human (PVB28) and murine (AK23) pathogenic anti-Dsg3 antibodies were able to dissociate the monolayers (2, 18) (Figure 4). In addition, PVF144 failed to synergize with a pathogenic antibody (Figure 4). These findings exclude a primary pathogenic function of anti-α-catenin autoAbs in PV, nevertheless a potential secondary role in the immunobiology of the disease cannot be excluded and warrants future studies.

## Discussion

In the present work we went forward in the characterization of the diverse targets of epithelium-specific autoreactive B cells from PV patients. To this purpose, we took advantage of the high-efficiency immortalization protocol of IgG+ memory B cells we had previously developed (10) and applied to isolate and molecularly characterize a number of anti-Dsg3 hMabs (2). Our present focus was to detect plasma-membrane-associated antigens, either expressed on the cell surface or intracellularly, therefore we chose to screen the isolated polyclonal cultures on both live and permeabilized keratinocytes. Our choice was based on the hypothesis that antigens associated to the cell membrane, even those with intracellular localization, could have a higher chance to contribute to pemphigus pathogenesis, which is due to the loss of cell-cell adhesion.

As expected, the vast majority of cultures resulted specific for Dsg1 and/or Dsg3, further confirming the major role of these antigens in pemphigus pathogenesis. Among polyclonal cultures that reacted only with permeabilized keratinocytes, we selected and cloned those giving a membrane-associated fishnet-like staining pattern on stratified epithelia. Among the cloned cultures, we further characterized clone PVF144 and demonstrated by IF, IP and IB studies that α-catenin is its target. α-catenin is a component of adherens junctions (AJs), i.e., cell-cell anchoring structures that, together with desmosomes, allow keratinocytes to adhere to one another and maintain epithelial integrity. α-catenin binds to β-catenin in AJs and is required for their formation and maintenance (19, 20). In addition, α-catenin was reported to be necessary for the organization of desmosomes in epithelial cells (21).

A previous study reported a reactivity of pemphigus sera to another AJ component, i.e., E-cadherin (22). Likewise, we showed that anti-α-catenin autoreactivity was: (i) well-represented in PV patient sera, as α-catenin was recognized by almost half of the PV sera tested; (ii) more frequently associated with pemphigus than with other autoimmune bullous diseases, as α-catenin was very rarely recognized by the BP sera analyzed.

Then, considering that previous studies demonstrated the ability of intact autoAbs to enter living cells (8, 16, 17), we addressed the potential pathogenicity of α-catenin-specific Mab PVF144 by evaluating its acantholytic activity in an *in vitro* keratinocyte dissociation assay. In this regard, several observations suggest that AJs, and in particular E-cadherin, may be involved in pemphigus pathogenesis (23, 24). Of note, with the exception of anti-desmocollin 3 autoAbs, all known non-Dsg-reactive autoAbs with reported pathogenicity do not possess acantholytic potential on their own but may act synergistically with anti-Dsg antibodies (1, 25, 26). Accordingly, Marchenko et al. described a pathogenic role for intracellular anti-mitochondrial autoAbs, even though not on their own (8). In our hands, the anti-α-catenin Mab PVF144 was not able to dissociate the keratinocyte monolayer either alone or in combination with a suboptimal dose of a pathogenic anti-Dsg3 antibody. Nevertheless, we cannot exclude that regions of α-catenin different from that recognized by PVF144 could be recognized by PV sera and contribute to acantholysis. In addition, a possible role of anti-α-catenin hMabs as co-factors in disease initiation or maintenance could not be exhaustively addressed by our approach. In previous studies, several antibodies against intracellular antigens have been considered as triggers for autoimmunity. Mabs specific for the cytoplasmic precursor form of Dsg1 (preDsg1) have been cloned from pemphigus patients and from healthy individuals (25, 26). Yamagami et al. postulated that the presence of anti-preDsg1 B cells is involved in the initiation of the autoimmune response in pemphigus patients. In particular, in the context of tissue damage they could present peptides which are part of mature Dsg1 (i.e., the extracellular autoantigen recognized by most pathogenetic autoAbs) derived from the processing of preDsg1. This intramolecular epitope spreading phenomenon could lead to the production of pathogenic autoAbs targeting mature Dsg1 and to the initiation of disease pathogenesis (27, 28). Moreover, natural autoAbs (NAAs), i.e., antibodies to intracellular autoantigens that naturally occur in the healthy population and are *per se* unable to cause immune phatology, have been theorized as cofactors in the onset of autoimmunity, possibly by participating in the mechanisms of chronic tissue injury at the basis of intermolecular epitope spreading (29–31).

In conclusion, while our previous study (2) demonstrated that pathogenic PV antibodies primarily target the Dsg-3 cis-interface, thus leading to desmosome disruption, our current results reveal novel antibodies targeting intracellular keratinocyte proteins. We are tempted to speculate that the cellular damage induced by pathogenic anti-Dsg antibodies may trigger an intermolecular epitope-spreading phenomenon resulting in an antibody response against intracellular antigens, among which α-catenin. Further studies are needed: (i) to evaluate whether PVF144 may act synergistically with anti-Dsg antibodies using more informative approaches, such as *in vitro* organ culture or *in vivo* models; (ii) to assess the possible role of anti-α-catenin autoAbs in pemphigus initiation and evolution *in vivo*; and (iii) to characterize this novel antigen as a possible target of epitope spreading phenomena in PV.

## Data Availability

The datasets generated for this study are available on request to the corresponding author.

## Ethics Statement

This study was carried out in accordance with the recommendations of Helsinki guidelines, IDI-IRCCS Ethics Committee. The protocol was approved by the IDI-IRCCS Ethics Committee. All subjects gave written informed consent in accordance with the Declaration of Helsinki.

## Author Contributions

GDZ and GDL have written the manuscript. GDZ, GZ, and AL have designed the study. GDZ, GDL, VC, and FM performed the experiments. All the authors have revised the manuscript and given the final approval for submission.

### Conflict of Interest Statement

The authors declare that the research was conducted in the absence of any commercial or financial relationships that could be construed as a potential conflict of interest.
