# Supplementary material for: Identification of a Novel Non-desmoglein Autoantigen in Pemphigus Vulgaris
Source: Front Immunol. 2019 Jun 19;10:1391. doi: 10.3389/fimmu.2019.01391 (PMC6593111; doi:10.3389/fimmu.2019.01391)
Supplement: Supplementary file 1 [file Image_1.pdf]

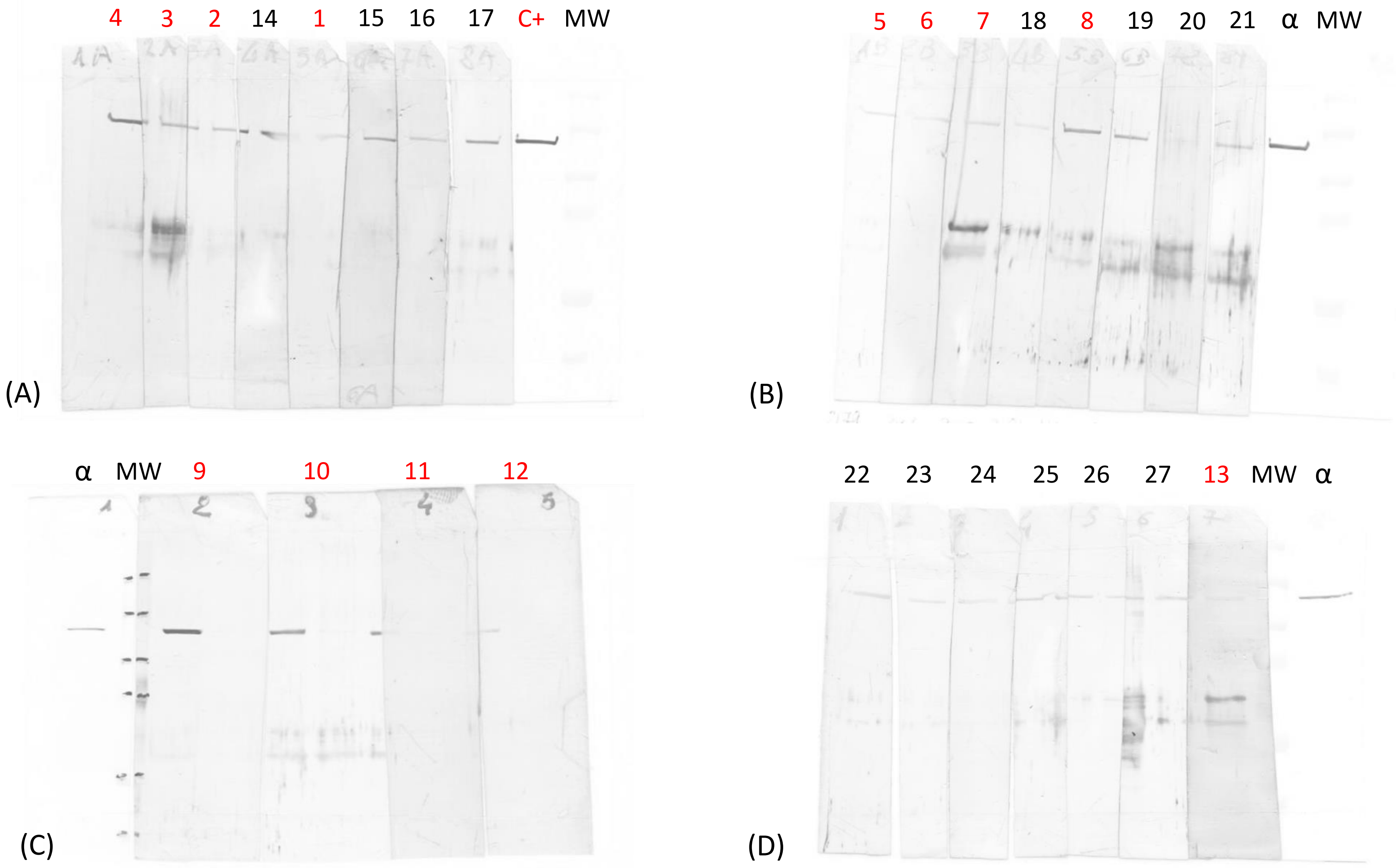

Quality control figure. PV sera specifically react with recombinant α-catenin. Raw data from immunoblotting experiments with sera obtained from 10 pemphigus vulgaris, 5 PNP and 6 NHI sera. Arabic numerales (in red) identify the same immunoblotting (IB) lanes indicated in Figure 3 and they refer to pemphigus vulgaris (PV) patients (1-10; Supplementary Figure 1A,B,C) and normal healthy individuals, NHI, (11-13; Supplementary Figure 1C,D); C+ is an anti-α-catenin antibody. MW is a prestained protein ladder. α refers to lanes incubated with the commercial anti-α-catenin antibody, not depicted in Figure 3. Lanes from 14 to 27, indicating serum samples not included in Figure 3, are listed below: 14: PNP (paraneoplastic pemphigus) 1; 15: PNP 2; 16: NHI 1; 17: PNP 3; 18: NHI 2; 19: PNP 4; 20: PNP 1; 21: PNP 5; 22: a repetition experiment with PV serum number 3; 23: PNP 3; 24: PNP 2; 25: PNP 4; 26: NHI 3; 27: PNP 5. In (C) positive control antibody (α) was analyzed on 150 ng of α-catenin, while serum samples were analyzed on 350 ng (left lane) and 150 ng (right lane) of protein. In all the other experiments 350 ng of recombinant α-catenin was loaded.
